# HPB News

**DOI:** 10.1155/1989/18745

**Published:** 1989


					HPB Surgery 1989, Vol. 1, p. 374-375
Reprints available directly from the publisher
Photocopying permitted by license only
1989 Harwood Academic Publishers GmbH
Printed in Great Britain
HPB NEWS
ANNUAL COURSE IN ADVANCED HEPATOBILIARY AND
PANCREATIC SURGERY
The Royal Postgraduate Medical School arranged its annual HPB Course on 6-10
November 1989 at the Wolfson Conference Center. The Course is organized by Mr
IS Benjamin and Professor RCN Williamson.
The topics covered
Investigation of liver and biliary tract primary and secondary liver tumours,
operative and non-operative management of gallstones, acute and chronic
pancreatitis, pancreatic cancer, pancreatic endocrine tumours, biliary strictures,
HPB trauma, liver transplant, technical aspects of HPB surgery with film and video
presentations and 'How I Do It' sessions.
The year's principal Guest Lecturer
Professor Henry A Pitt, Johns Hopkins Hospital, Baltimore, USA
Other guest speakers
LH Blumgart (Switzerland), J Buckels (Birmingham), MJ Cooper (Exeter), J
Farndon (Bristol), FT Gorey (Dublin), PA Grace (Dublin), J Hermon-Taylor (St
George's), KEF Hobbs (Royal Free), I Ihse (Sweden), CW Imrie (Glasgow), GAD
McPherson (Wycombe), GJ Poston (St Mary's), MC Puntis (Cardiff), B Ringe
(Germany), K Rolles (Royal Free), BJ Rowlands (Belfast), RCG Russel (The
Middlesex Hospital), H Saeger (Germany), R Williams (King's College) in addition
to the organizers and teachers from Royal Postgraduate Medical School.
Information can be obtained from:
Wolfson Conference Centre
Royal Postgraduate Medical School
Du Cane Road
London W12 0NN
Telephone: International 44-1. 740 3117
374
HPB NEWS 375
AUSTRIAN CHAPTER OF THE WORLD ASSOCIATION OF HPB
SURGERY
The Austrian Chapter had its annual business meeting on May 26 at the Congress
Center in Graz. The members elected were
Dozent U. Wayand, Linz, as the national president,
Dr O. A. Weitensfelder, Klagenfurt, as national secretary, and
Professor Funovics, Vienna, as national delegate.

				

